# Correction to “Assessment of PLA Depolymerization for Circular Economy: Production Pathways, Physical Properties, Thermodynamics, and Kinetic Modeling”

**DOI:** 10.1021/acs.iecr.6c00336

**Published:** 2026-02-05

**Authors:** Adam McNeeley, Y. A. Liu

In Figure 16, in the top two rows of chemical reactions under Straight Chain, the reaction type for the top row should be esterification, not transesterication; and the reaction type for the second row should be transesterication, not esterification. The corrected figure appears below:

**Figure 16:**
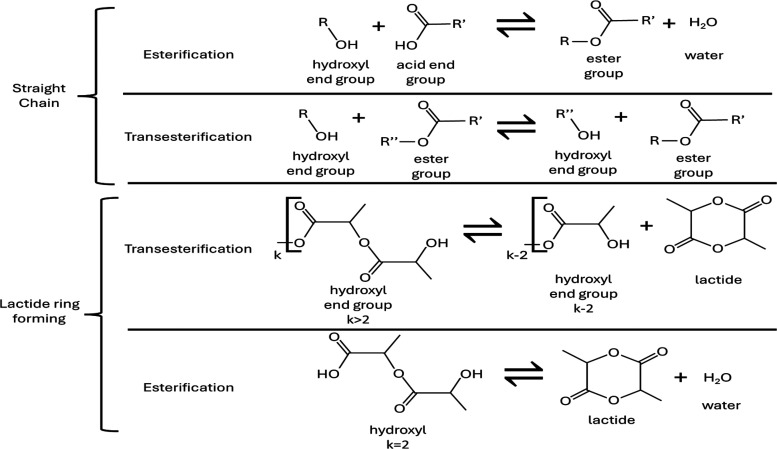
Summary of the four primary reactions relevant in PLA and lactic acid systems.

